# Optimizing multi-parameter distributed fiber sensors: a hybrid Rayleigh-Brillouin-Raman System approach

**DOI:** 10.1038/s41377-024-01392-7

**Published:** 2024-02-05

**Authors:** Kwang Yong Song

**Affiliations:** Dept. of Physics, Chung-Ang University, 84 Heukseok-ro, Dongjak-gu, Seoul, 06974 Korea

**Keywords:** Fibre optics and optical communications, Optical sensors

## Abstract

An optimized single-end hybrid Rayleigh, Brillouin, and Raman distributed fiber sensing system has been developed for simultaneous measurement of multiple parameters. This system integrates 3-bit pulse coding for the Raman signal and the Brillouin amplification of the Rayleigh-backscattered signal, discriminating strain, temperature, and vibration using a single sensing fiber.

## Abstract (web summary)

Hybrid Rayleigh-Brillouin-Raman distributed sensing system: Coded pulse pairs are employed for simultaneously measuring vibration, strain and temperature distributions, through an optimized detection scheme of Rayleigh, Brillouin, and Raman scatterings.

## Graphical abstract

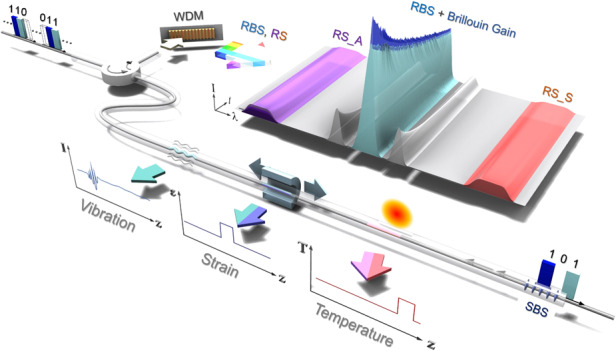

A distributed optical fiber sensor (DOFS) is an intrinsic sensor that is able to determine the spatial distribution of one or more measured parameters (or measurands) at each and every point along a sensing fiber. Today, DOFS has gained widespread usage, primarily for real-time monitoring of the structural integrity of expansive civil infrastructures and surveying environmental conditions^1^. These sensors serve as fundamental components in smart sensing systems, playing a crucial role in the development of smart cities and the implementation of smart factories equipped with automated manufacturing processes.

DOFS are capable of measuring various physical quantities such as strain^2^, temperature^3,4^, pressure^5^, vibration^6^, acoustic impedance^7^, and more. These sensors operate utilizing elastic or inelastic light scatterings within optical fibers, which are Rayleigh backscattering (RBS), Brillouin scattering (BS), and Raman scattering (RS). In most applications, strain, temperature, and vibration are the primary variables of interest, and each type of scattering exhibits distinct sensitivity to these measurands. For example, RS, involving scattering by optical phonons, exclusively responds to temperature change making it ideal for implementing distributed temperature sensor (DTS), and BS, relying on scattering by acoustic phonons, are sensitive to both strain and temperature. Meanwhile, RBS, arising from the random fluctuation of refractive index, is particularly suitable for measuring vibration, enabling the realization of distributed acoustic or vibration sensor (DAS / DVS).

The utilization of multiple scatterings presents a direct approach to building DOFS capable of simultaneously measuring multiple measurands. This has prompted researchers to propose diverse hybrid systems^8,9^. One such example involves combining RBS and BS as the foundation for a hybrid system aiming to detect vibration concurrently with strain or temperature. In these systems, Brillouin optical time domain analysis (BOTDA) or reflectometry (BOTDR) is employed to measure strain or temperature, while phase-sensitive optical time domain reflectometry (phase-OTDR or *ϕ*-OTDR) is utilized for vibration measurement^8^. However, a significant challenge hindering the practical application of this hybrid system is the cross-sensitivity of strain and temperature observed in both RBS and BS signals. While integrating an RS-based DTS system into the hybrid setup could potentially offer a solution for discriminating between strain and temperature variations, it significantly amplifies the complexity of the system.

In the work newly published in *Light: Advanced Manufacturing*^10^, the research team led by Xinyu Fan and Zuyuan He at Shanghai Jiao Tong University proposed a new approach for optimizing hybrid DFOS systems. This method leverages the combined use of RBS, BS, and RS signals while employing a single-end measurement configuration. In the proposed system two optical pulses with slightly different optical frequencies are injected into a sensing fiber, where the RBS signal from the initial pulse is amplified by the subsequent pulse through Brillouin amplification by stimulated BS (SBS; Fig. 1).

**Fig. 1:**
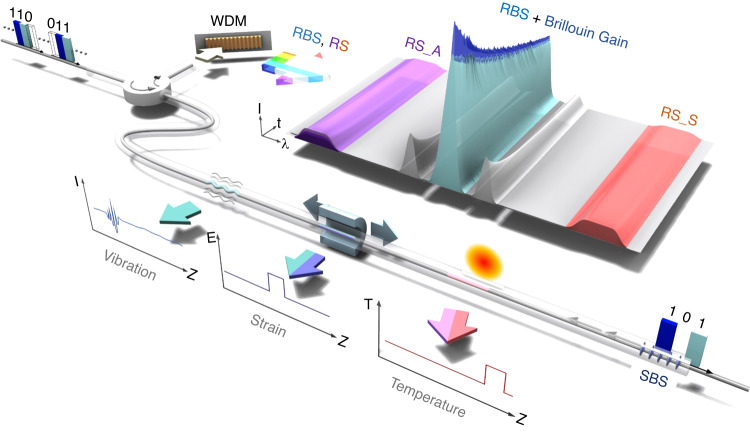
Schematic of a hybrid Rayleigh-Brillouin-Raman distributed sensing system. Coded pulse pairs (101, 110, 011) are injected. Rayleigh backscattering (RBS) of the 1st pulse serves dual purposes: vibration sensing and probing BOTDA via SBS with the 2nd pulse. Stokes (RS_S) and anti-Stokes (RS_A) Raman scattering of the pulse pair are employed for DTS after the decoding process. A wavelength-division multiplexing (WDM) filter is used for selective detection of RBS, RS_A, and RS_B

In general, BOTDA utilizing the SBS between counter-propagating light waves offers the advantage of generating larger signal amplitudes compared to BOTDR which involves spontaneous BS. However, BOTDA necessitates access to both ends of the sensing fiber, limiting its practicality in various applications. The method utilizing RBS as the probe for BOTDA was recently developed by the team of Fan and He^11^, and has also been applied for this work. This approach effectively merges the RBS and BS measurements, ensuring a substantial signal amplitude through SBS while maintaining a single-end access configuration to the sensing fiber. Furthermore, this method simplifies signal processing in contrast to earlier hybrid RBS and BS systems^8,9^, as it solely employs the RBS light wave for demodulating the probe for both BS and RBS.

For the implementation of DTS a 3-bit simplex coding method is employed for encoding the two successive optical pulses. Given the optical frequency offset between the two pulses for SBS (~10 GHz) is negligible compared to the spectral width of RS signal (several THz), two optical pulses can be assumed to have an identical frequency for generating RS. While various pulse coding methods have been utilized to improve the signal-to-noise ratio (SNR) in long-range DOFS systems^3,12^, it is noteworthy that the pulse coding strategy employed in the proposed hybrid system not only enhances the SNR, but also maintains the spatial resolution of DTS to the level of single pulse width when operating with two successive pulses for RBS-BS scheme.

The measurement accuracies demonstrated by the developed hybrid system are as follows: $10\,{\rm{p}}{{\varepsilon }}{{/}}\sqrt{{\rm{Hz}}}$ for *ϕ*-OTDR, 0.58 MHz for BOTDA, and 0.5 °C for DTS achieved with a spatial resolution of 10 m within a sensing range of 9 km. These reported accuracies align with those of conventional DOFS systems operating within similar sensing ranges. While certain independent system schemes can achieve superior performances, they often necessitate additional schemes or devices, consequently increasing system complexity and cost. For instance, conventional BOTDA requires two-end access to the sensing fiber. In contrast, the proposed hybrid system achieves multi-parameter sensing while fully isolating each parameter with a single-end access configuration to the sensing fiber.

The peak power of optical pulses used in the proposed system must be maintained lower than that of conventional DTS systems due to limitations imposed by detrimental nonlinear effects on BS, which poses challenges in reducing the spatial resolution. Further enhancement in the sensing range and the spatial resolution without significantly compromising the system’s simplicity are anticipated, potentially through the application of advanced scheme like distributed amplification^13^.

In conclusion, the proposed system offers a significant reduction in system complexity and cost compared to three sets of conventional independent systems. The pivotal elements in effectively merging the RBS, BS, and RS measurements with a single light source involve utilizing RBS as the probe for BS and implementing the pulse coding technique for RS. The developed approach appears particularly suitable for long-distance distributed sensing applications that demand simultaneous measurement of multiple parameters, such as real-time and long-term health monitoring of rail transit systems.
